# Transmission is a Key Driver of Resistance to the New Tuberculosis Drugs

**DOI:** 10.1056/NEJMc2404644

**Published:** 2025-01-02

**Authors:** Galo A. Goig, Chloé Loiseau, Nino Maghradze, Kakha Mchedlishvili, Teona Avaliani, Daniela Brites, Sonia Borrell, Rusudan Aspindzelashvili, Zaza Avaliani, Maia Kipiani, Nestani Tukvadze, Levan Jugheli, Sebastien Gagneux

**Affiliations:** Swiss Tropical and Public Health Institute, Allschwil, Switzerland; National Center for Tuberculosis and Lung Diseases (NCTLD), Tbilisi, Georgia; Swiss Tropical and Public Health Institute, Allschwil, Switzerland; National Center for Tuberculosis and Lung Diseases (NCTLD), Tbilisi, Georgia; Swiss Tropical and Public Health Institute, Allschwil, Switzerland

To the Editor

Multidrug-resistant tuberculosis (MDR-TB) is a growing public health problem^1^. Recently, the World Health Organization endorsed new regimens for the treatment of MDR-TB that rely on new and repurposed antibiotics bedaquiline, pretomanid, linezolid with or without moxifloxacin (BPaL(M))^2^. Since BPaL(M) is used to treat MDR-TB, and much of the global burden of MDR-TB is driven by patient-to-patient transmission of drug-resistant *Mycobacterium tuberculosis* (Mtb)^3^, resistance to BPaL(M) drugs emerging in MDR-TB patients could spread. On the other hand, experimental studies have shown that resistance to even a single drug can reduce the fitness of Mtb^4^, and because most Mtb strains acquiring resistance to BPaL(M) are already resistant to many first- and second-line drugs, such strains might be less transmissible. Yet, the contribution of patient-to-patient transmission to the growing burden of BPaL(M) resistance has not been determined.

Here we analyzed the genomes of a 13-year nationwide collection of 6,926 clinical Mtb isolates from the country of Georgia, a known hotspot of MDR-TB^5^. We identified a total of 60 Mtb strains that were resistant to at least isoniazid, rifampicin (i.e. the most important first-line drugs), fluoroquinolones, and one of the other BPaL(M) compounds; henceforth, we refer to these strains as ‘highly drug-resistant’. Drug resistance was inferred using WGS data, not by phenotypic testing. Excluding mixed infections, we found that 16/58 (28%) of these highly drug-resistant strains belonged to one of four genomic clusters. Within each of these clusters, all strains exhibited an identical mutational profile, indicating patient-to-patient transmission (Figure 1). To determine if transmission of highly drug-resistant strains also occurs in other settings, we analyzed an additional 81,576 Mtb genomes from global sources. We identified a further 454 highly drug-resistant strains in 26 countries. Excluding mixed infections, we found that 117/420 (28%) of these strains occurred in one of 41 genomic clusters, involving strains from 10 countries in the Americas, Europe, Africa and Asia. Additionally, we identified nine highly drug-resistant strains from four countries that carried resistance mutations to all BPaL(M) compounds, thus potentially classifying as “totally drug-resistant” (See Appendix for further details).

Our results show that, despite BPaL(M) being introduced recently, resistance to these new TB drugs has already developed in at least 27 countries across four continents. Furthermore, a quarter of these cases involved patient-to-patient transmission. Our findings call for improvements in diagnostic capacity, infection control and surveillance, without which, the longevity of BPaL(M) is at risk.

A complete list of author names is available with the full text of this letter at NEJM.org.

Supported by the Swiss National Science Foundation (grants 320030-227432, and CRSII5_213514) and the European Research Council (883582-ECOEVODRTB).

Disclosure forms provided by the authors are available with the full text of this letter at NEJM.org.

## Supplementary Material

Supplementary Appendix

Supplementary Appendix

## Figures and Tables

**Figure 1:**
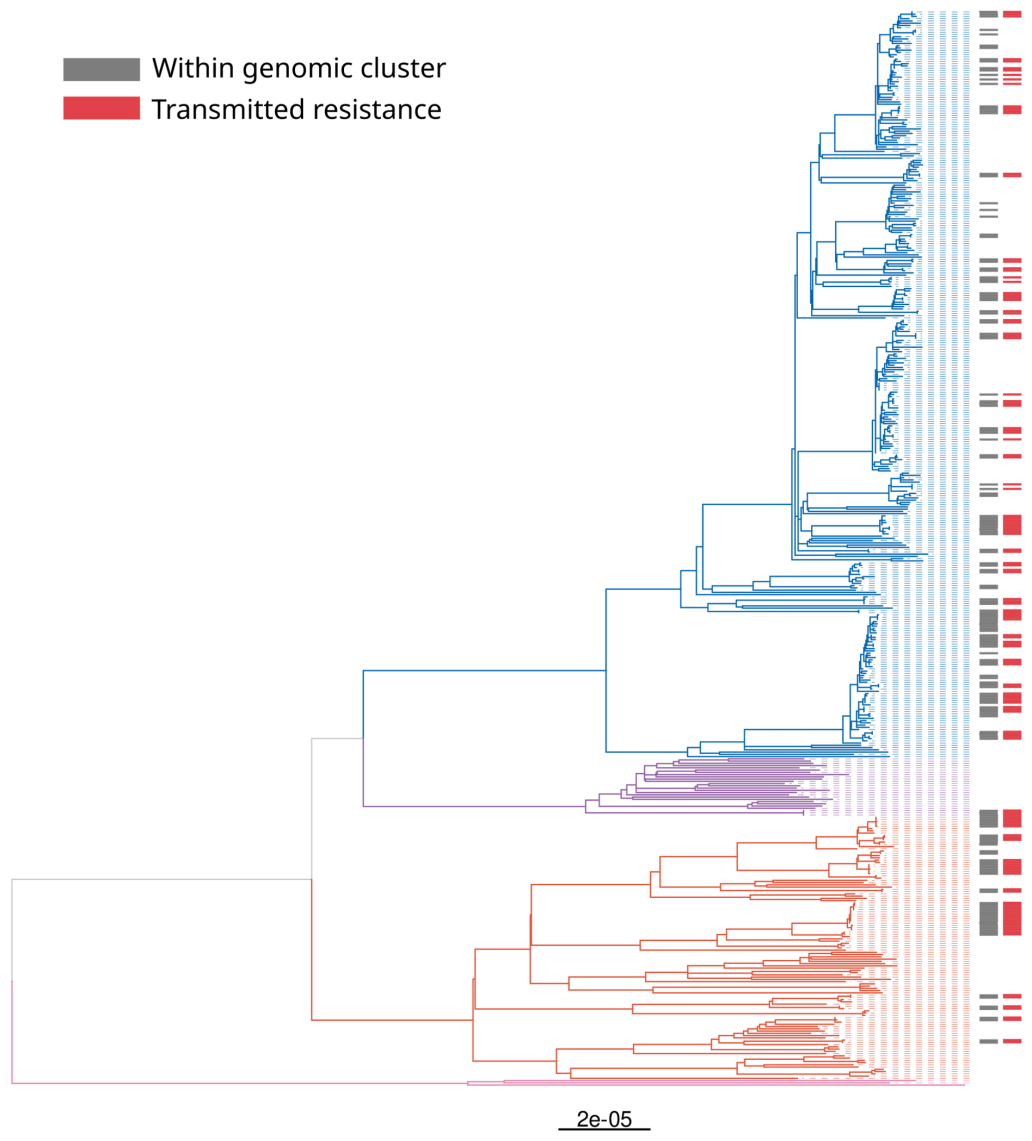
Maximum likelihood-phylogeny of highly drug-resistant Mtb strains identified in this study. The tree scale indicates substitutions per site per genome. Branches are coloured according to the MTBC lineage: pink for lineage 1, blue for lineage 2, purple for lineage 3, and red for lineage 4. Discrete heatmaps next to the phylogeny indicate whether the strains were within a genomic cluster (gray), and whether they shared the same drug resistance-conferring mutations, in which case transmitted resistance was inferred (red).
